# Topic: Distribution of *Anopheles stephensi* bioforms in selected districts of Rajasthan, India

**DOI:** 10.1371/journal.pone.0313227

**Published:** 2025-02-21

**Authors:** Sangeeta Singh, Robin Marwal, Suman Lata, Poonam Saroha, Sanjeev Kumar Gupta, Himmat Singh

**Affiliations:** 1 ICMR-National Institute of Malaria Research, New Delhi, India; 2 Academy of Scientific and Innovative Research (AcSIR), Ghaziabad, India; 3 National Centre for Diseases Control, New Delhi, India; Central University of South Bihar, INDIA

## Abstract

**Introduction:**

*Anopheles stephensi* is a major urban malaria vector in Rajasthan, India, and is responsible for spreading persistent malaria throughout the year. In Rajasthan, *An*. *stephensi* is invariably distributed and has three bioforms discriminated based on the number of the ridge on the eggs viz; *Type*, *Mysorensis*, and *Intermediate*. The present study aimed to understand the distribution pattern of these bioforms in rural and urban setups as they also have differences in their malaria transmission capacity.

**Methods:**

Gravid mosquitoes and the larvae were collected from different habitats of districts of Rajasthan. The gravid females *An*. *stephensi* were allowed to lay eggs. These eggs then were subjected to morphometric analysis and counted for the number of ridges for bioform identification.

**Results:**

About 15000 ± 200 eggs were obtained from ~190 gravid *An*. *stephensi* collected from 45 localities (11 Urban & 34 Rural) of eight districts of Rajasthan. Out of which 3569 eggs were subjected to morphometric analysis. Mysorensis bioform (49.7%) was observed to have higher percent over Intermediate (25.5%) and Type (24.6%) bioforms. Mysorensis and Intermediate were found more in rural areas whereas the Type bioform dominated higher in urban areas.

**Discussion:**

The Mysorensis bioform was found to be dominant throughout the year in all seasons in rural areas. Type bioform preferred indoor breeding places like underground tanks, cement tanks whereas other bioforms preferred outdoor breeding places. Egg size was found to be directly proportional to the number of ridges on the eggs (r = 0.55). No reproductive isolation was observed among these bioforms.

**Conclusions:**

The Mysorensis bioform is more prominent than other bioforms. Subspecies level understanding helps to plan effective control measures according to the breeding site selection majorly by Type bioform, an efficient vector in this region.

## Introduction

Malaria affects half of the world’s population and about 87 countries are at risk of infection. In 2022, approximately 249 million cases and more than six million deaths were reported [1]. According to the World Health Organization (WHO) Malaria Report 2023, the Southeast Asian Region contributes approximately 2% of the global burden of malaria, with India being the major contributor (65.2%) of this region [1]. In India, the five states i.e., Odisha, Chhattisgarh, Maharashtra West Bengal, and North-eastern states have contributed to about 85–90%, and Rajasthan accounted for 1–2% of the total malaria cases reported in India between 2012 and 2022 [2].

Anopheles is responsible for malaria in several countries across the world [1]. In India, out of 58 reported Anopheline species, only six are major vectors i.e. *Anopheles culicifacies*, *Anopheles fluviatilis*, *Anopheles minimus*, *Anopheles baimaii*, *An*. *epiroticus and An*. *stephensi* for malaria transmission [3,4]. *Anopheles stephensi* contributes to about 12% of malaria in India.

Although *Anopheles culicifacies* transmit malaria in rural parts of the country and contribute about 65% of cases annually and also maintain seasonality of malaria transmission and responsible for outbreaks [3]. *Anopheles fluviatilis* is responsible for the transmission of malaria (15%) in the plains and foothills [5]. *Anopheles minimus*, and *An*. *baimaii* causes malaria in the forest regions of the northeastern states of the country, and *An*. *epiroticus* transmits malaria in the Andaman and Nicobar Islands, which is a brackish water species [6]. The origin of *An*. *stephensi* Liston 1901 is Oriental region and in India, it is considered a major urban malaria vector. *An*. *stephensi* is responsible for persistent malaria transmission throughout the country except for Andaman and Nicobar Islands, however, scanty reports from North-East states of India [7]. The global presence of *An*. *stephensi* and its significant role in spreading malaria make it crucial for epidemiological study. Additionally, its surveillance and control are essential for comprehensive global disease control efforts [8,9].

Malaria vectors, other than *An*. *stephensi* have been characterized as species complexes with some morphologically indistinguishable sibling species which vary in their role in malaria transmission. Whereas *An*. *stephensi* still is not classified as sibling species but rather as bioforms. Although these are morphologically indistinguishable, there are no significant physiological and molecular distinct [6–8]. The distribution of *An*. *stephensi* is now global [10,11], and studies have shown that the species has expanded its geographic presence in various regions of the world such as Africa, the Middle East, and Sri Lanka, and has adapted to different urban environments [12,13]. In the decade between 2012 and 2023, *An*. *stephensi* invaded Djibouti (2012), Ethiopia (2016), Sudan (2016), Sri Lanka (2017), Somalia (2019), Nigeria (2020), Yemen (2021), Kenya (2022), Eritrea (2022), Ghana (2023) and these invasions are potentially linked with anthropogenic activities including construction, urbanization, transportation systems, and availability of suitable habitats (S1 Fig) [1,3,8,10,11,13–17].

In the Rajasthan state of India, *An*. *stephensi* has been reported as the primary malaria vector in both urban and rural areas [18–20]. In rural areas of western Rajasthan, *“Tanka”* and “*Beri”* are water-storing underground tanks, used due to scarcity of water [21,22]. These *tankas* and wells provide favorable breeding and resting environmental conditions to *An*. *stephensi* which likes to breed in them throughout the year. Moreover, *An*. *stephensi* finds favorable conditions for both breeding and resting within houses, which helps to skip the exposure to extreme environmental conditions such as heat and dryness.

Some studies in the past have highlighted that *An*. *stephensi* exists in more than one biological form and referred as bio-forms, variants, eco-variants, or sub-species [13,23,24]. The existence of two bioforms i.e. “Type” and “Mysorensis” was reported by Knowles and Basu (1934) [25]. Subsequently, Ramsay and McDonald (1936) and Mulligan and Baily (1936) expressed the possibility of the existence of two bioforms [26,27]. Sweet and Rao (1937) [12] exhibited significant differences in certain egg measurements of these two bioforms. Knight et. al. (1959) listed *An*. *stephensi* variety Mysorensis in ‘Catalog of the Mosquitoes of the World’ [15,28]. Subbarao *et al* [24] reported another bioform of this species with an egg ridge number 13–16 and designated it as an ‘Intermediate’ bioform. In India and Iran, this species exists in three biological forms viz. ‘Type’, ‘Intermediate’, and ‘Mysorensis’ [15,21,24,28]. Among these three bioforms, the Type bioform is reported to be an efficient vector of urban malaria, whereas, Mysorensis and Intermediate are considered to be rural and poor vectors [10,29].

All three bioforms of *An*. *stephensi* can only be differentiated based on egg morphological characters i.e., the number of ridges on the egg floats as there are no other morphological characteristics based on which the bioforms can be differentiated at the adult stage. The Type bioform has the highest egg ridges 16–22, whereas, the Mysorensis and Intermediate bioforms have 10–14 and 15–16 egg ridges respectively [12]. There is no comprehensive study on *An*. *stephensi* bioforms in the past 20 years by Nagpal et.al. (2003) [30].

Considering *An*. *stephensi* is a major malaria transmitting vector, there is a need to understand its distribution pattern and occurrence of different bioforms as they are reported to have different vectorial capacity. In the past taxonomic studies based on morphological characters and geographical distribution were done by Christopher in 1933 and documented in the records of "*Fauna of British India*"(6,7), the ‘Taxonomic Keys for Mosquitoes’ by Puri (1931) [31] and in ‘Anophelines of India’ by Rao 1984 [6]. Thereafter, no study on bioforms of *An*. *stephensi* taxonomy is comprehensively done. Therefore, the present study aims to gather information on the distribution of *An*. *stephensi* bioforms and their association with malaria transmission, focusing on their identification and distribution pattern in urban and rural areas of Rajasthan from 2020 to 2023.

## Methodology

### Study area

The Indian state of Rajasthan is located (27.0238° N and 72.2179° E) in the western part of the Indian subcontinent with spread of 342,239 km^2^, that covers about 10.4% of India’s total geographical region. Rajasthan also shares its international border with Pakistan and with other Indian states i.e. Gujarat, Madhya Pradesh, Haryana, Punjab, and Uttar Pradesh. Rajasthan has about 69% of the "*Thar*" desert, also known as the Great Indian Desert, which is the easternmost extension of the vast Sahara Desert (measuring 2,85,680 km^2^). The state’s geography includes different ecological systems, including the desert in the west, the Aravalli hills, and the sub-humid region in the east. The arid part of Rajasthan receives less than 20 cm of rainfall per annum, with high-temperature extremes ranging from 0 degrees Celsius (November-January) to 50 degrees Celsius (May-June). On the other hand, the Aravalli hills receive between 50–100 cm of rainfall per annum, with relatively lower temperature extremes [32]. These extreme environmental conditions have a direct bearing on the prevalence of mosquitoes [33].

**Fig 1:**
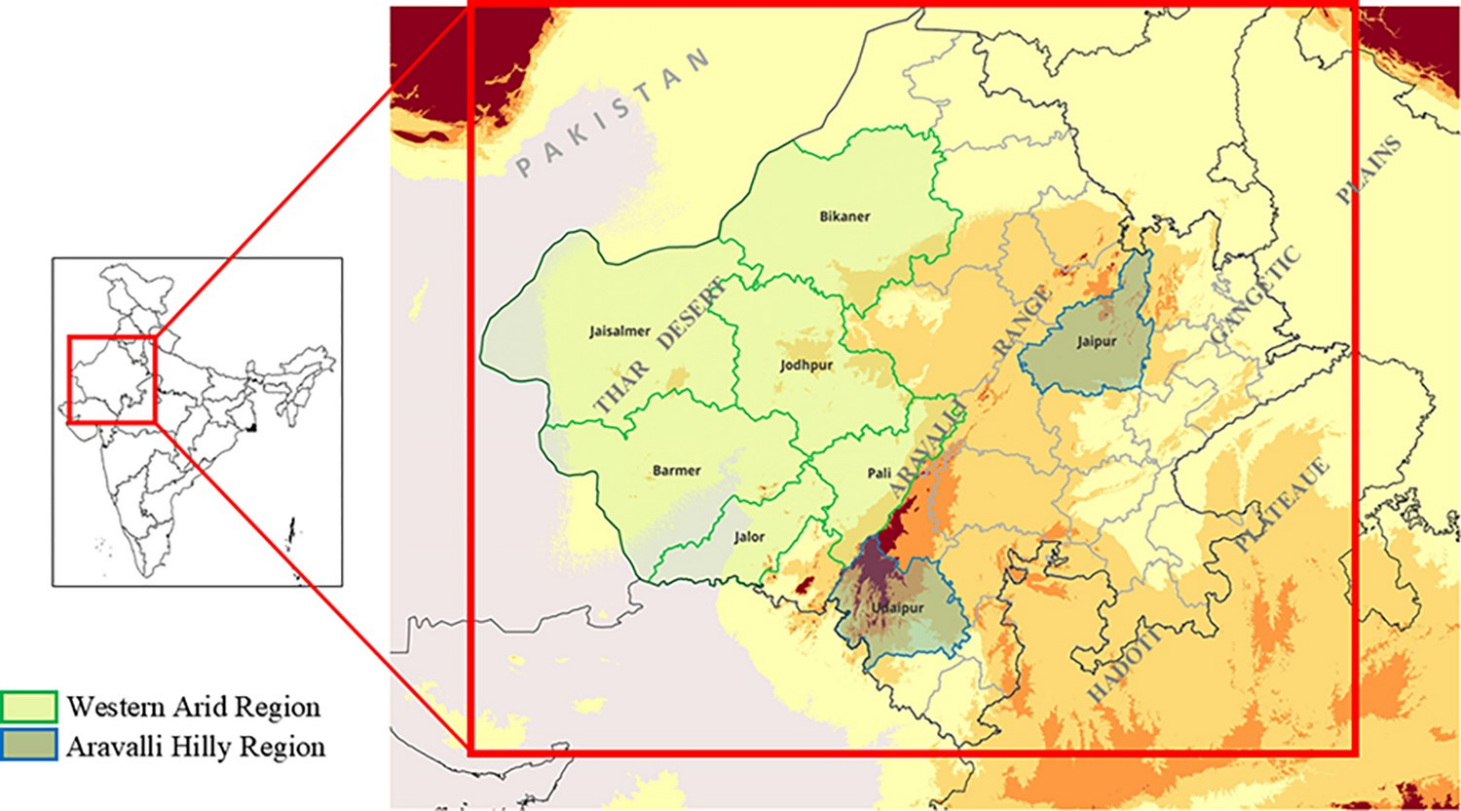
Map illustrates the study areas in Rajasthan, distinguishing between the Western arid region, which includes districts such as Jalore, Jodhpur, Barmer, Bikaner, Jaisalmer, and Pali, known for the Thar Desert, and the Aravalli hilly region comprising Jaipur and Udaipur.

The study was carried out in urban and rural localities of 8 districts of Rajasthan situated in the western arid region (Jalore, Jodhpur, Barmer, Bikaner, Jaisalmer, and Pali) and Aravalli hilly region (Jaipur and Udaipur) (Fig 1).

### Adult mosquito collection

*Anopheles* habitats were surveyed 2–4 km around the villages and different locations for adults and larvae in different seasons for three years (2020–2023). Adult mosquitoes were collected at dawn and dusk (6:00–8:00 a.m.; 7:00–8:00 p.m.) from cattle sheds and houses [34]. Adult mosquitoes were collected using the hand catch method with aspirators and torches in separate collection cloth cages (9 x 9 x 9 cm size), and transported to the laboratory. Species identification of Anopheles was done using standard taxonomic keys, *An*. *stephensi* were separated and transferred to labelled cloth cages (12 x 12 x 12 cm size) for ovipositioning [5–8]. Individual field-collected gravid *An*. *stephensi* were allowed to lay eggs in separate bowls. About 10–15 eggs from each bowl were randomly picked up for morphometric observations under a compound microscope (40x) and after counting their ridges the egg laying female *An*. *stephensi* was classified into different subspecies. The collection sites of the mosquitoes were enumerated such as cattle sheds and human dwellings (such as bedrooms, living rooms, and bathrooms), and spaces like under stairs both in urban and rural settings. Information on the resting surface of the adult mosquitoes like cement walls, mud walls, wooden surfaces, hanging clothes, and roofs was gathered to understand the habitat preferences across different bioforms.

### Larval collection

Larvae were searched and collected from all the habitats where the potential of larvae breeding could occur such as ponds, cement tanka, underground tanks, ditches, and plastic containers in the study sites. The collected larvae were reared into adults and the species were identified using the standard identification key [5–8]. To identify the bioform in the larval habitats these collected larvae obtained from different habitats were reared to adults separately and allowed to mate in different cages and allowed to lay eggs after blood meal in the laboratory condition i.e. 27±2°C and humidity 70%± 5%. The eggs were then morphologically identified based on ridge counts. Habitat preference was estimated on the basis of the F1 generation of the larvae collected from different habitats. We presented qualitative results of all bioform breeding preference in this communication.

### Eggs ridge count and morphometric analysis

**Fig 2:**
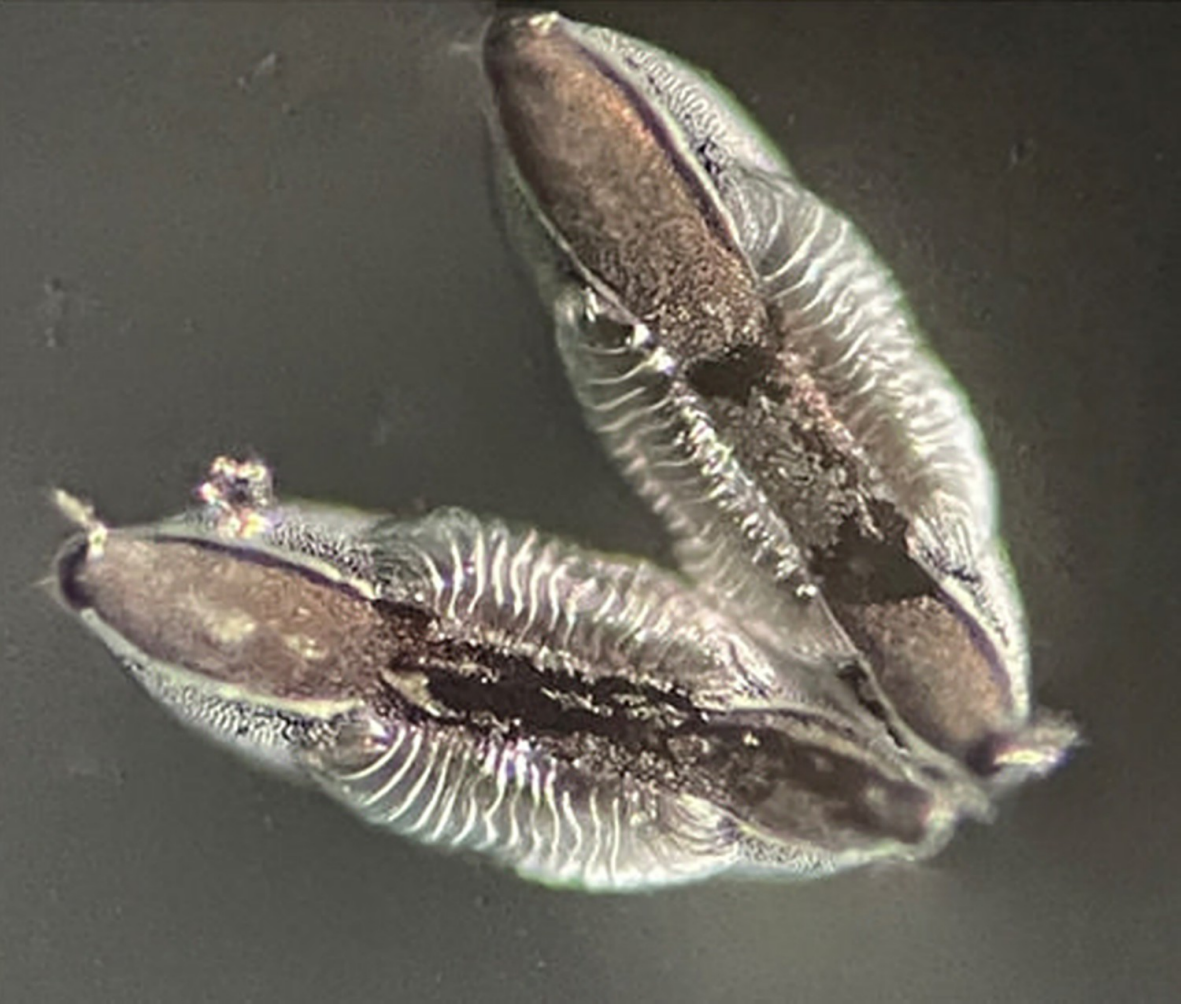
An image of *Anopheles stephensi* eggs, showcasing floats and ridges, was captured by a microscope at 10x and 40x magnifications. Wild gravid females were allowed to lay eggs, and subsequently, the egg ridges were counted and measured using an Occulo-meter. In this figure, 19 ridges were observed and counted under the microscope which is a ‘Type’ bioform.

For morphometric analysis, the length, breadth, and number of ridges on the egg float were measured under a compound microscope (Zeiss stem 2000-c) at 10x and 40x magnification using an occulo-meter. The occulo-meter, was calibrated against a stage micrometer at 10x by superimposing the lines of the occulo-meter with a stage micrometer (10μm each division), and the number of ridges along one side of the egg float was counted. Each egg was measured three times under microscope to avoid focal bias out of three readings the reading that appeared tice was taken as correct reading whereas measurement of 10–12 eggs of isofemales were done to observe the mean values (Fig 2). The area of an egg was also calculated to understand any relation of an area with the egg arrangement on the water surface and the number of ridges. A formula for the calculation of area of elliptical area was used [35]. Area = π x transverse radius of egg x longitudinal radius of egg which is equal to the working formula (Considering the egg as an elliptical object.) as follows:

$$\mathrm{Areas}=\pi \;x\;½\;\mathrm{width}\;\mathrm{of}\;\mathrm{egg}\;x\;½\;\mathrm{length}\;\mathrm{of}\;\mathrm{egg}\;({\mathrm{mm}}^{2})$$

Similarly, the arrangement of the eggs (ovipositional venation on the surface of the water) on the water surface was visualized first and then the number of ridge counts was done and recorded to get information related to egg arrangements and Type of bioform differentiation of different bioforms of *An*. *stephensi*.

All three bioforms of *An*. *stephensi* were categorized based on egg morphological characters i.e., the number of ridges on the egg floats. The eggs were categorized as Type bioform if egg ridges were between16-21, eggs bearing ridge count between 15–16 were categorized as Intermediate whereas, the eggs with ridge count between 10–14 considered as Mysorensis bioform respectively [12,29]. Iso-female lines of different bioforms were also reared in insectary conditions and eggs were obtained [34]. The eggs were counted and allowed to hatch and the percent hatching was calculated. Each gravid female was categorized based on the number of ridges on the eggs laid by them.

The crossbreeding experiments of bioforms, Mysorensis, and Type were conducted to understand reproductive isolation and to assess mating success in a controlled environmental condition in the laboratory at 27±2°C and humidity 70%± 5%. The mosquitoes collected from Isofemale lines of Type and Mysorensis were allowed to mate using different combinations, i.e., 4 male mysorensis and 2 female Type bioform and vice versa (4 males Type /2 females Mysorensis) (S1 Table). The experiment was conducted only once to evaluate the crossbreeding between two bioforms.

### Data analysis

The distribution pattern analysis of field-collected *An*. *stephensi* was done using Microsoft Excel. The morphometric data of eggs was analyzed for length, ridges and area using one-way ANOVA (Analysis of Variance) in SPSS software (version 16) and x Chi-square and F test were used for test of significance for comparing egg morphometry of different bioforms. The map was prepared using Q GIS [36].

### Ethics statement

This study was carried out as a part of an ongoing project (PHB/NIMR/EC/2020/199) with the approval of the Institute Ethics Committee ICMR- National Institute of Malaria Research, Sector 8, Dwarka, New Delhi.

## Results

About 15,000 ± 200 eggs were obtained from ~362 gravid *An*. *stephensi* from 45 localities (11 Urban and 34 Rural) of 8 districts of Rajasthan, with 3569 eggs subjected to morphometric analysis. Mysorensis bioform was prominent (49.7%) followed by Intermediate (25.5%) and Type bioform (24.6%) (Table 1). The egg ridges exhibited considerable variation, encompassing a wide range extending from 9 to 23. The majority of the total egg sample (n = 3569), approximately 90%, fell within the range of 12 to 18 egg ridges very few eggs had low ridges from 9–11 and high ridges ranged from 22–23. It was observed that the egg ridges of the Mysorensis bioform were in the range between 9 and 14, whereas in the Intermediate bioform, this range was between 15 and 16, and the Type bioform has a range between 17 and 23. The most prevalent egg ridge range was found to be between 13 and 14, representing 38% of the sample, followed by 15 to 16, which accounted for 35.5% of the eggs (S2 Table).

**Table 1:** *Anopheles stephensi* bioforms distribution on the basis of egg ridge counts from the different study districts of Rajasthan.

Districts	Locality	No. of Gravid Females	Mysorensis	Intermediate	Type	Total
**Barmer**	Urban	24	20	78	151	249
	Rural	120	548	293	315	1156
**Bikaner**	Urban	2	0	4	16	20
	Rural	32	91	70	167	328
**Jaipur**	Urban	8	34	24	16	74
**Jaisalmer**	Urban	1	7	0	0	7
	Rural	34	132	122	99	353
**Jodhpur**	Urban	88	575	233	33	841
	Rural	31	279	23	20	322
**Pali**	Urban	1	6	8	0	14
	Rural	1	12	0	0	12
**Jalore**	Rural	6	2	19	41	62
**Udaipur**	Urban	14	69	39	23	131
**Total Rural**		**138**	**1064**	**527**	**642**	**2233**
**Total Urban**		**224**	**711**	**386**	**239**	**1336**
**Total**		**362**	**1775**	**913**	**881**	**3569**

**Fig 3:**
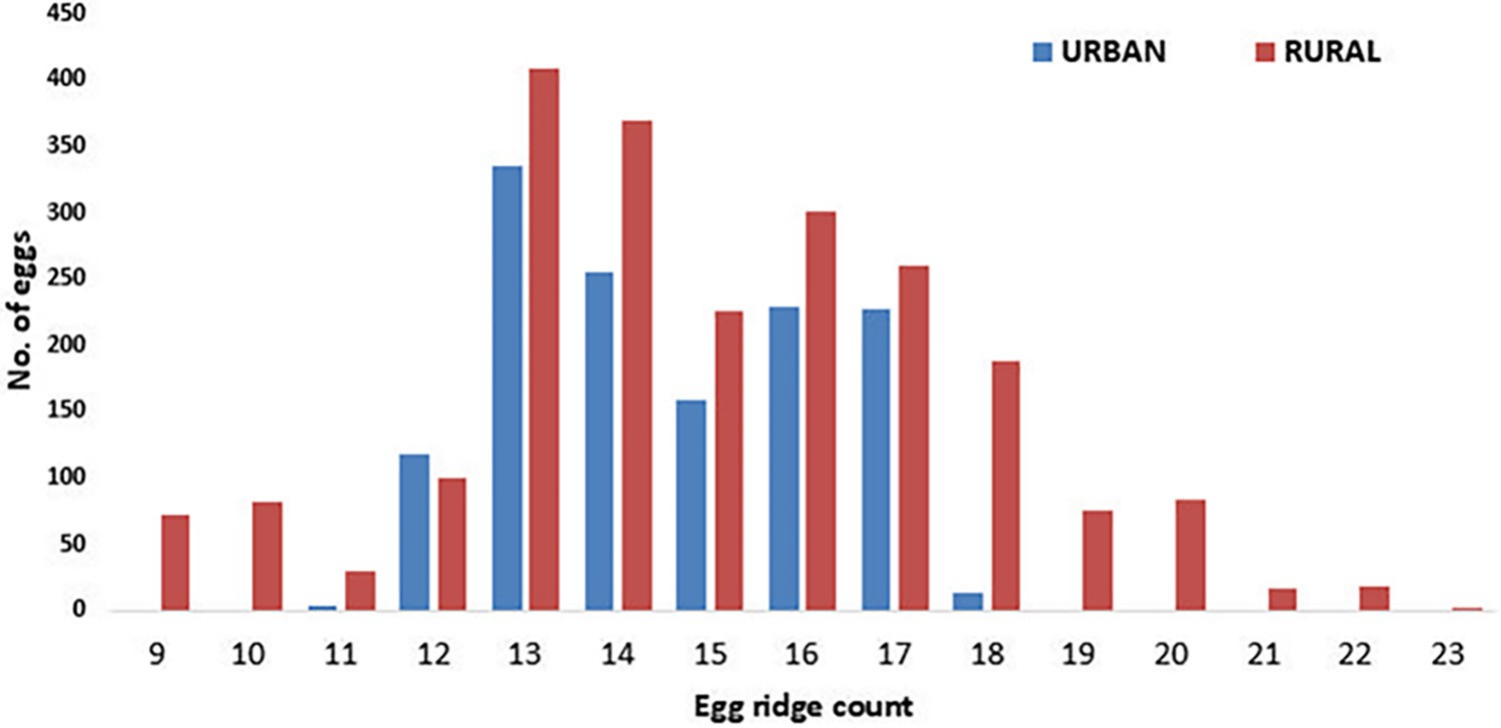
The distribution of ridge counts found in *Anopheles stephensi* eggs across urban and rural areas in Rajasthan.

There was a distinct difference in the range of egg ridge counts between urban and rural areas in Rajasthan, with a total sample size of 3569 eggs. In urban settings, the range was notably constrained, spanning from 11 to 18 egg ridges. Conversely, in rural areas, the range extended from 9 to 23, indicating a broader variation in egg ridge counts within this context (Fig 3).

**Fig 4:**
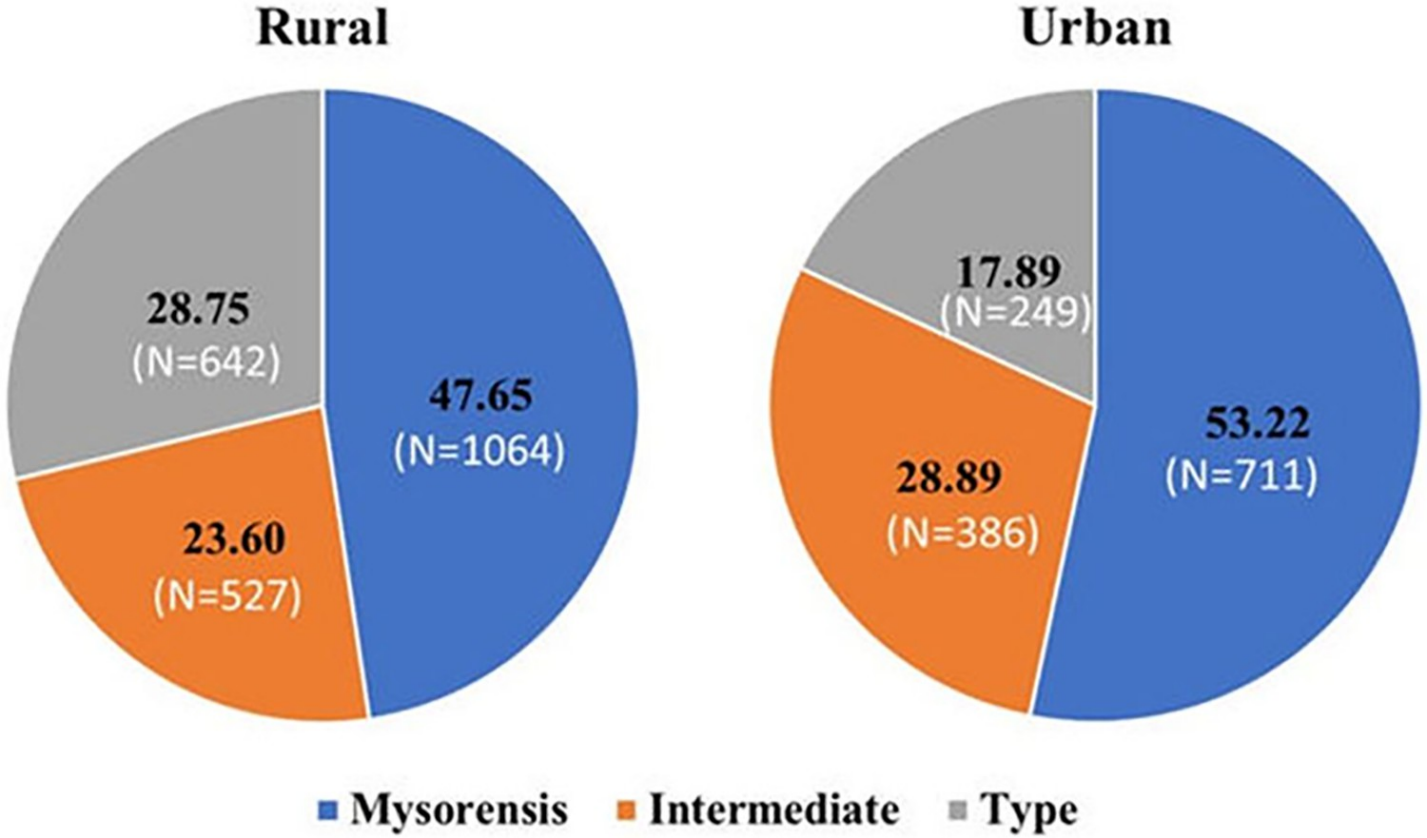
The percent distribution of bioforms in rural and urban areas of Rajasthan; Mysorensis bioform exhibits the highest prevalence in both rural and urban settings, followed by the Intermediate, and then the Type bioform.

As compared to Type and Intermediate bioforms, the presence of Mysorensis was significantly higher (50%) in both rural (48%) and urban (53%) settings. It was observed that *An*. *stephensi* Mysorensis (53.22%) and Intermediate (28.89%) were found more in rural areas (Fig 4). The Type bioform was significantly higher in urban areas (χ^2^ = 47.69, p<0.00001, df = 5).

**Fig 5:**
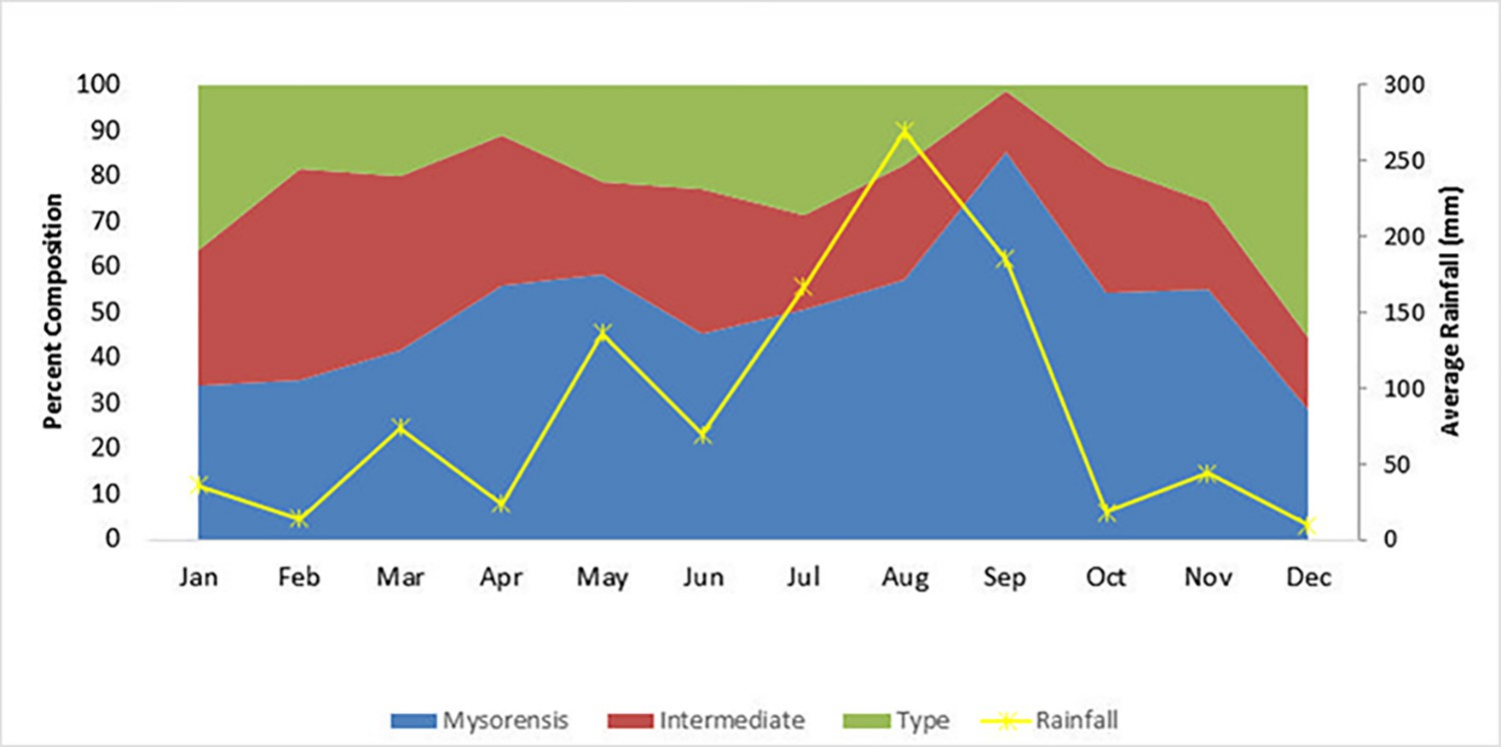
The monthly variation in the composition of *Anopheles stephensi* bioforms in Rajasthan, establishes a clear connection with rainfall. The spikes in the graph not only explain the relationship between the mosquito composition and rainfall but also highlight the influence of the one-month lag period of rainfall on mosquito population dynamics.

The percentage of Mysorensis was more than double throughout the year except for a few months of winter (December to March) irrespective of their urban or rural habitats. In August and September, the composition reached about 85%. The increase in Type bioform composition was found to overlap with the malaria transmission season (October-December) in Rajasthan. It was also observed that the population size of Intermediate bioform was higher during January-July (Fig 5). Rainfall showed a positive correlation (r = 0.58) of a percent increase in the composition of Mysorensis. However, the correlation was not strong but the other bioforms showed a negative correlation with Intermediate (r = -0.401) and Type (r = -0.307) respectively. The population of Mysorensis was found to be significantly higher than the Type population of the region.

F1 population obtained from the emergence of collected larvae from water bodies, house premises, and containers, indoors and outdoors, showed a preference for bioform breeding, it was observed that Type bioform preferred cemented tanks and underground tanks (*Tankas*) that are mostly indoors. On the other hand, bioform Mysorensis was found mostly in outdoor cattle tanks, pond ditches, outdoor cemented tanks, outdoor wells, and also in turbid water. Intermediate bioform was found in overhead tanks and cattle tanks that are outdoors similar to Mysorensis bioform (Table 2).

**Table 2:** Breeding habitats of different bioforms of *Anopheles stephensi* in urban and rural localities in eight selected districts of Rajasthan from 2020 to 2023.

Locality	Type	Intermediate	Mysorensis
Urban	Ground tank Underground Tanks Indoor Cement Tanks, Plastic, Clay Pots	Overhead tanks Underground Tanks	Cement Tank, Cattle Tanks, Ditches, Pond
Rural	Plastic Containers, Coolers Cemented Tanks, Cattle Tanks, Bird Pots	Ground cattle tanks, Ditches, Pond	Pond, Plastic Containers, Cement Tanks, Cattle Tanks, Community Water Storage

**Fig 6:**
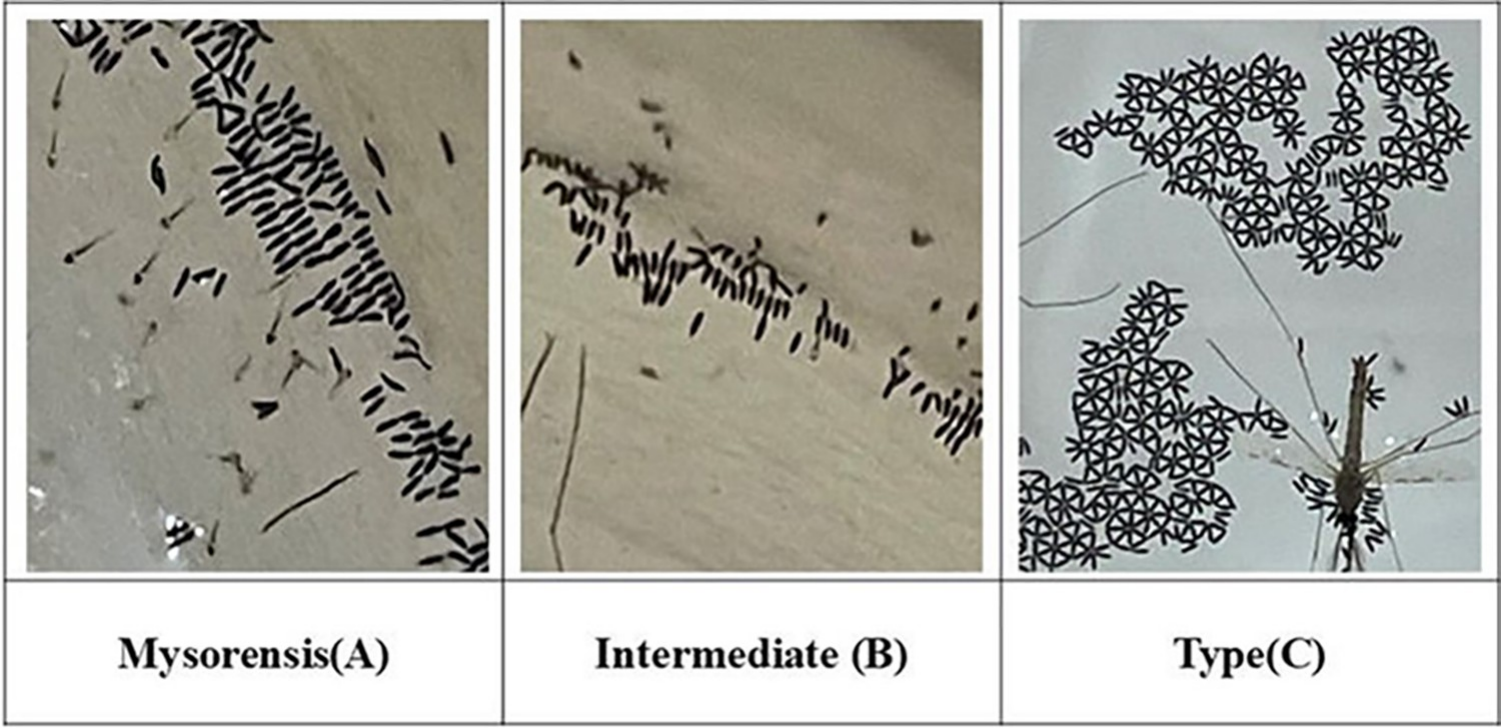
The egg arrangement of Anopheles bioforms exhibits distinct patterns. In Mysorensis (A) and the Intermediate bioform (B), eggs are arranged linearly, whereas in the Type bioform (C), eggs are arranged in a rosette pattern.

The eggs laid on water surface were observed to be laid in some pattern. The eggs laid in a linear arrangement were from the Mysorensis and Intermediate bioforms (Fig 6A and 6B) having lower ridge counts. Whereas, the eggs with high ridge counts (Type bioform) were laid in a rosette formation (Fig 6C).

The Crossbreeding experiments of bioforms Mysorensis and Type showed no reproductive isolation. The isofemale of Mysorensis laid 75.5 (average of 2 females) with variable egg ridges ranging from 14 to 18 when allowed to mate with males from Type iso-female line with 90.7% emergence and 82% survival till adult formation. Similar results were obtained when isofemale of Type bioform were allowed to mate with males obtained from the isofemale line of Mysorensis bioform (Average eggs 88.5; ridge range 13–18; and 80% pupation).

Gravid females of both bioforms (n = 20 each) were allowed to lay eggs individually, revealing that the Type bioform lays significantly more eggs (75±5.16) than the Mysorensis (65±2.79), with the egg-laying capacity of the Type bioform being significantly higher (χ^2^ = 7.39, p≥0.025).

**Fig 7:**
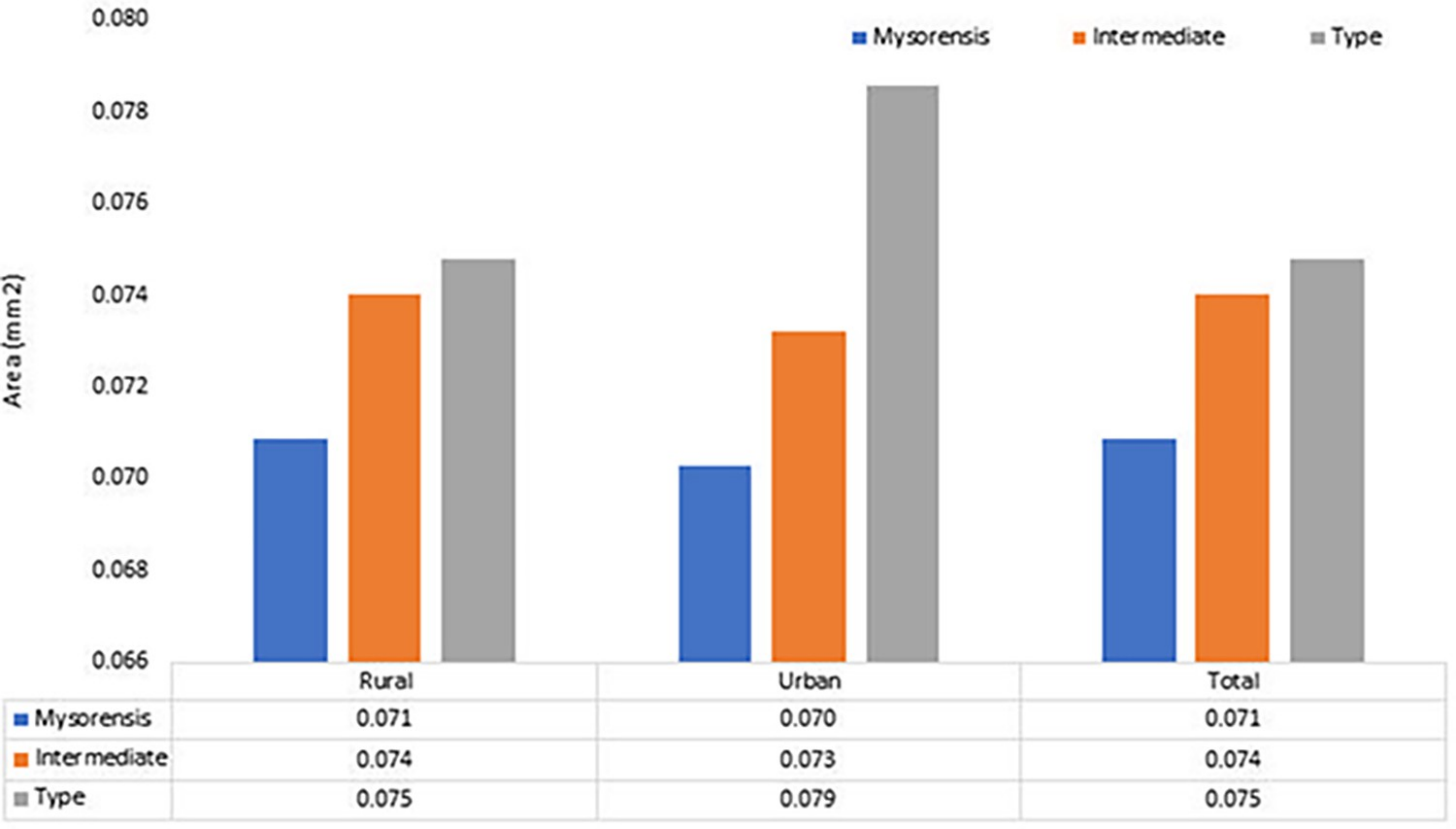
Comparison of average egg areas among various bioforms of *Anopheles stephensi* across urban and rural habitats (n = 3569); Type bioform exhibits the largest average egg area of 0.075mm^2^, followed by the Intermediate bioform 0.074mm^2^, and the Mysorensis bioform with the smallest average egg area of 0.071mm^2^.

Average areas of eggs were compared between, bioforms, and Type was found to be significantly bigger area (p< 0.001) than other bioforms. The size of eggs was larger collected from rural areas than in urban localities although the difference was not statistically significant. This difference in size was found in all bioforms (Fig 7).

On the other hand, hatchability was highest in Mysorensis bioform followed by Type (76%) and Intermediate (72%) Type, the difference was not significant (χ^2^ = 2.64 at df = 2 for Mysorensis) (Table 3).

**Table 3:** Laboratory observations on the *Anopheles stephensi* life cycle of Type and Mysorensis bioforms.

Bioforms (Total number of eggs)	Avg eggs laid/ female (±SD)	Percent Hatching
Mysorensis (n = 516)	64.5 ± 1.9	96
Intermediate (n = 506)	72.2 ± 3.1	72
Type (n = 606)	75.7 ± 4.0	76

Overall parameters among the eggs from the gravid mosquitoes collected from rural locations were larger than those collected from urban areas. The average egg length of Type bioform was larger (550.8±0.028μm) than Intermediate (533.0±0.023μm) and Mysorensis (515.0±0.025μm). This consistent trend of size differences was also observed in the surface area of the eggs, with the bioform Type demonstrating significantly larger surface areas. (Table 4).

**Table 4:** Egg parameters of *Anopheles stephensi* bioforms collected from rural and urban locations.

Locality	Parameters	Mysorensis	Intermediate	Type
Rural	Length(μm^2^) ± SD (Range)	517.5± 26.9 (600–412.6)	536.41±25 (634.8–416.6)	552.6±30 (666.6–454.9)
	Width (μm^2^) ± SD (Range)	174.96± 11.6 (211.6–125)	176.82±20.3 (222.1–125)	168.83±19.8 (211.6–125)
	Area (mm^2^) ± SD (Range)	0.07086± 0.006 (0.0967–0.045)	0.0740±0.008 (0.15–0.040)	0.074± 0.010 (0.1055–0.045)
	Ridge± SD (Max-Min)	12.89±1.26 (9–14)	15.57±0.49 (15–16)	18.17±1.32 (17–23)
Urban	Length (μm^2^) ±SD (Range)	511.7±22.7 (454.9–581.9)	529.3± 20.6 (476.1–603)	545.9± 23.6 (486.6–634.8)
	Width(μm^2^) ± SD (Range)	174.6± 11.6 (148.1–211.6)	175.8± 11.0 (158.7–211.6)	182.8±14.0 (158.7–222.1)
	Area (mm^2^) ± SD (Range)	0.0703± 0.0062 (0.059–0.094)	0.0731± 0.0062 (0.0593–0.1055)	0.0785± 0.0086 (0.064–0.1055)
	Ridge± SD	13.187 ± 0.7 (11–14)	15.59±0.49 (15–16)	17.05± 0.21 (17–18)
Total	Length(μm^2^) ± SD (Range)	515.2±24** (412.6–600)	533.4±23** (416.7–634.8)	550.8±28.7** (454.9–666.7)
	Width (μm^2^) ± SD (Range)	174.8±11.6 (125–211.6)	176.4±17.0 (125–222.18)	172.6±19.5 (125–222.18)
	Area (mm^2^) ± SD (Range)	0.0708± 0.0068** (0.040–0.105)	0.0740± 0.0086** (0.045–0.096)	0.0747± 0.0100** (0.0450–0.1055)
	Ridge± SD	12.89±1.26 (9–14)	15.57± 0.49 (15–16)	17.86 ±1.23 (17–23)

**Significantly different at p< 0.001 through F test.

Table 4 shows the egg morphometry, including the average length, width, and area of bioforms. The average length of bioform Type was found to be significantly higher than other bioforms (F = 576.8; P≤0.001. Similarly, the mean egg areas of the bioforms also showed significant differences (F = 124.7, P≤0.001).

## Discussion

Malaria is more prominent in rural areas than in urban areas of Rajasthan [37]. Mysorensis bioform is said to be prominent in rural which our study also confirms. However, some studies suggest that Mysorensis is a zoophilic and a poor vector [38]. Intermediate and Mysorensis bioforms were mainly found in rural settings in and around Delhi [23]. Our study also revealed similar findings for the distribution of Mysorensis. The Intermediate bioform was comparatively higher in the rural areas, while the Type bioform was dominant in urban areas. However, the difference was not significant, suggesting that a mixed population of bioforms occurs in both urban and rural areas depending on the availability of breeding sites. A similar distribution was observed by other studies conducted in the extreme western regions of Baluchistan, and Iran [16,39].

*Anopheles stephensi*, mysorensis bioform was reported to be dominant in Sri Lanka [40]. Our field collections also showed that there are seasonal variations in the distribution of different bioforms, Mysorensis bioform remained the predominant species during the post-monsoon season where outdoor breeding was predominant this also coincides with transmission season in Rajasthan. Additionally, during the monsoon and post-monsoon season (July to November), Mysorensis bioform co-exists with the seasonal *An*. *culicifacies*. All bioforms occur in both rural and urban locations of Rajasthan similar to the other studies conducted earlier [41]. Interestingly, although the breeding sites were not isolated to any specific bioform, the Mysorensis bioform was primarily found in outdoor shallow water bodies (Table 4). The selection of breeding habitat also depends upon larvivorous predators. Studies have reported that the presence of predators like Gambusia/Copepods/ Tadpoles deter oviposition by airborne mosquitoes as these predators regulate mosquito populations and even influence their oviposition and habitat-selection behavior [42].

The egg of *An*. *stephensi* is shiny jet-black in color and has a ‘skiff‐shaped’ appearance with its anterior end slightly broader than the posterior end [43]. Type bioform eggs were found significantly larger than other bioforms (Table 4). The largest average egg length was observed in a study conducted in Sri Lanka as mentioned in Table 5.

**Table 5:** Egg morphological characteristic of *Anopheles stephensi* biological forms Rajasthan as compared to other studies in the past.

Biological form	Egg Details	Rajasthan	Hormozgan, Iran [16]	Baluchistan [39]	Fars (Kareron), Iran [39]	Pakistan [44]	India, (Delhi) [24]	South of Iran [41]	India [12]	Srilanka [40]
**Mysorensis**	No. of eggs examined	1775	129	3870	NA	1472	1030	452	6916	470
	Mean No. of ridges ±SD (* = mode)	12.89±1.26	13.6±1.30	12.55±0.73	NA	13.87±1.15	10–14*	12.05	13.50±1.25	12–14*
	Length (μm) Mean +SD	515±0.0254	468.88±19.40	470.60±27.00	NA	482.83±19.13	NA	448.5	477.12±23.87	580±0.5
	Ridge width (μm) Mean +SD	170±0.0116	225.2±23.70	198.00±18.2	NA	217.58±20.30	NA	231	218.08±20.44	240±0.2
**Intermediate**	No. of eggs examined	913	81	120	120	NA	989	NA	NA	381
	Mean No. of ridges ±SD (* = mode)	15.57±0.49	15.4±0.89	15.11±1.27	15.11±1.27	NA	13–16*	NA	NA	15–16*
	Length (μm) Mean +SD	533.78±22.38	542.10±18.50	544.00±8.07	544.00±8.07	NA	NA	NA	NA	570±0.9
	Ridge width (μm) Mean +SD	170±0.0170	255.8±13.80	248.20±6.74	248.20±6.74	NA	NA	NA	NA	230±0.2
**Type**	No. of eggs examined	881	513	1472	NA	NA	667	NA	4707	149
	Mean No. of ridges ±SD (* = mode)	17.86± 1.23	18.7±1.70	13.87±1.15	NA	NA	16–19*	NA	17.82±1.55	17–19*
	Length (μm) Mean +SD	550.8±0.0287	596.52±24.70	482.83±19.13	NA	NA	NA	NA	548.06±26.59	590±0.5
	Ridge width (μm) Mean +SD	173.0±0.019	314.8±24.24	217.58±20.30	NA	NA	NA	NA	289.65±22.25	250±0.2

The average length of Mysorensis eggs was found to be larger than the average egg length of Mysorensis studied in the rest of India, Pakistan, and Iran.

The length of eggs corresponds to the number of ridges on the eggs and showed positively correlated (r = 0.55) (Table 4) i.e., higher the ridge number means larger the length of eggs.

Type species distribution was restricted and contributed about 23.62% of composition in the overall distribution of the bioforms in Rajasthan. The percent composition of Type and Mysorensis populations was observed dynamic and kept on changing during different seasons during summers (April–June) the percent composition of Type bioform was increased in all the urban and rural settings (Fig 4). This may be attributed to the fewer outdoor breeding sites available in the dry season to Mysorensis and the Type bioform is adapted to underground tanks and indoor habitats. (Table 4)

The transmission capacity of mosquitoes is also dependent on their anthropophilic behavior and the presence of the cattle population which may provide zooprophylaxsis. A study on *An*. *stephensi* in southern Iran by Vatandoost *et*. *al*. (2006) [16] also concluded that the transmission capacity depends on the availability of cattle in the rural areas due to the presence of bioform Mysorensis which is known as zoophilic and considered as a poor vector [24]. However, in western Rajasthan rural areas, our study showed the presence of Type bioform which is considered an efficient vector due to its high anthropophilic index and high transmission rates despite the cattle population in rural areas. A recent study in Iran suggests that Mysorensis has the potential to survive long enough to be re-infected and transmit *Plasmodium vivax* malaria several times and has transmission capacity at par with other vector species [45]. which is evident in western Rajasthan as well where *Plasmodium vivax* malaria is prominent. The distribution of Type bioform in rural areas needs more investigation, especially during hot summers.

The higher percentage of hatching of Mysorensis eggs may be attributed to the high competition in outdoor breeding species than mostly container breeder-like Type bioforms more study is needed to explain the egg viability and hatching. The Type showed less hatching ability than other bioforms, despite breeding in safe places like tanks drums and underground tankas.

In our controlled laboratory study it was found that Mysorensis and Type bioform mate easily in caged conditions (under laboratory conditions of 27±2°C and humidity 70%± 5%) we put both conditions using male Mysorensis versus female Type *An*. *stephensi* (4 males ‘Mysorensis’ /2 females ‘Type’) and vice versa and found successful mating and emergence, similar findings were shown by Rutledge *et al*(1970) [23] who did not find any hybrid sterility in the crosses between bioforms laboratory strains derived from Iran. Our results of the cross-breeding experiment showed that there is no reproductive isolation between *Anopheles stephensi* bioforms however, this was observed that the F1 generation obtained by these experiments suggests that the egg ridges also change with cross-breeding i.e. if the Type female was crossed with Mysorensis male they tend to lay eggs with lower egg ridges than the Type bioform. The adults obtained from the cross did not show any sterility and reduction in egg numbers (egg laid /female) when they were allowed to mate among themselves. Our study showed Mysorensis prefers larger ponds, and wetlands of fresh water more a peridomestic habitat with open locations whereas the Type habitat is more in small cemented containers, underground tanks (*tankas*) and they prefer peridomestic to indoor breeding as well in coolers cattle tanks and underground tanks (Table 2). The Type was found to lay more eggs than Mysorensis which may be attributed to the indoor breeding habit where competition with other mosquito species is comparatively less and the survival of mosquitos is higher in indoor climatic conditions than those breeds outdoors. However, more studies need to be done to find the egg-laying capacity of these bioforms.

Based on egg length correlation with the number of ridges on eggs it was seen that if the length of an egg increases there is an increase in ridge count (r = 0.5570, weak correlation), in rural (r = 0.565) the correlation was lower than in urban (r = 0.6347). The high number of ridges supports larger eggs which may be similar to the larger boats requiring more water displacement for floating on water. There is a need to have an in-depth study of the environmental conditions of *An*. *stephensi* of bioforms as it is observed that there is a change in ridge numbers in laboratory conditions after a few generations of captivity this might be an environmental alteration and may be the larval food, competition, blood feeding to adult females, etc. Some molecular studies and Spiracular indices [30] as identifying tools could not find a significant difference in the bioforms although these two races of *An*. *stephensi* were given sub-species status by Puri [13] and this was accepted by Stone et al [28], which needs an in-depth study of ecological variation whether to keep species biotype or ecotype only. Studies conducted by Alam et.al. (2008) & Mishra et.al. (2021), found no significant difference between ribosomal DNA ITS2 and D-3 domains and the *An*. *stephensi* odorant binding protein (AsteObp1), in the two sub-variants of Mysorensis and Type, as observed by Gholizadeh et. al. [46–49].

Although there was no clear-cut isolation in the breeding habitats of bioforms but Type bioform was exclusively found in indoors and underground tanks called “*Tankas*”. They were also adapted to low light conditions, on the other hand, Mysorensis was prevalent in seepage water and distributed away from human dwellings. These breeding area selections might be responsible for the slight difference in bioform isolation and making them reproductively isolated in sympatric conditions. Further studies on niche selection and isolation of ecotypes of *An*. *stephensi* are necessary to evaluate the differential distribution of bioforms in the region. The mating experiment suggested that, given the opportunity, they can interbreed, an in-depth study on breeding isolation on these bioforms is required. Studies have also shown that the distribution of *Anopheles stephensi* is increasing in different continents along with all its bioforms, suggesting co-habitation of all bioforms indoor and outdoor habitats (Sri Lanka, Africa [1,3,8,10,11,13–17]. Studies on the distribution of bioforms may provide evidence of uneven malaria incidences in similar mosquitogenic systems. However, this study has certain limitations i.e. Scanning Electron Microscope for surface structure studies of eggs and molecular identification on the eggs for bioform identification was not conducted. Additionaly, the cross-breeding experiment was conducted only once.

## Conclusion

Mysorensis bioform was dominantly found in both urban and rural areas. The percent composition of Type bioform was more in the rural than in the urban areas. The distribution of *An*. *stephensi* bioforms depends on the availability of preferred breeding habitat i.e. Type bioform was highly associated with indoor and large container breeding, while the Mysorensis bioform was associated with natural water bodies such as ditches and ponds.

This study revealed that bioforms are not reproductively isolated. While the number of egg ridges, the key for bioform identification, is correlated with the size of the egg. This finding can be used for the selection and implementation of control interventions efficiently. Further study is warranted to understate the epidemiological impact of different bioforms and the impact of habitat availability on the distribution of bioforms. The Type bioform of *An*. *stephensi* in both rural and urban might be playing a crucial role in the persistent transmission of malaria in the region. The findings of this study can be used by the state control program and the National Vector-borne Diseases Control Program of the country.

## Supporting information

S1 Fig The timeline of the distribution and invasion into different countries illustrates the spread of *Anopheles stephensi* from its native countries to various countries from 2012 in Asia, the Middle East, and Africa. This chronology provides specific details about the expansion of *Anopheles stephensi*, tracing its journey from its origin and highlighting its invasion into different countries in Asia, the Middle East, and Africa.
(DOCX)

S1 Table Crossbreeding Experiment between *Anopheles stephensi* bioforms. (DOCX)

S2 Table Morphometric analysis of *An*. *stephensi* bioform egg ridges from different locations. (DOCX)

S3 Table Excel file of raw data of the study. (XLS)
