# Correction: Distribution of *Anopheles stephensi* bioforms in selected districts of Rajasthan, India

**DOI:** 10.1371/journal.pone.0331939

**Published:** 2025-09-08

**Authors:** Sangeeta Singh, Robin Marwal, Suman Lata, Poonam Saroha, Sanjeev Kumar Gupta, Himmat Singh

The word “Topic:” is incorrect in article title. The correct title is: Distribution of Anopheles stephensi bioforms in selected districts of Rajasthan, India. The correct citation is: Singh S, Marwal R, Lata S, Saroha P, Gupta SK, Singh H (2025) Distribution of Anopheles stephensi bioforms in selected districts of Rajasthan, India. PLoS ONE 20(2): e0313227. https://doi.org/10.1371/journal.pone.0313227
