# Supplementary material for: Topic: Distribution of Anopheles stephensi bioforms in selected districts of Rajasthan, India
Source: PLoS One. 2025 Feb 21;20(2):e0313227. doi: 10.1371/journal.pone.0313227 (PMC11844831; doi:10.1371/journal.pone.0313227)
Supplement: S1 Fig — This chronology provides specific details about the expansion of Anopheles stephensi, tracing its journey from its origin and highlighting its invasion into different countries in Asia, the Middle East, and Africa. (DOCX) [file pone.0313227.s001.docx]

**S1 Figure :** The timeline of the distribution and invasion of *Anopheles stephensi* from its native countries to various countries from 2012.

**
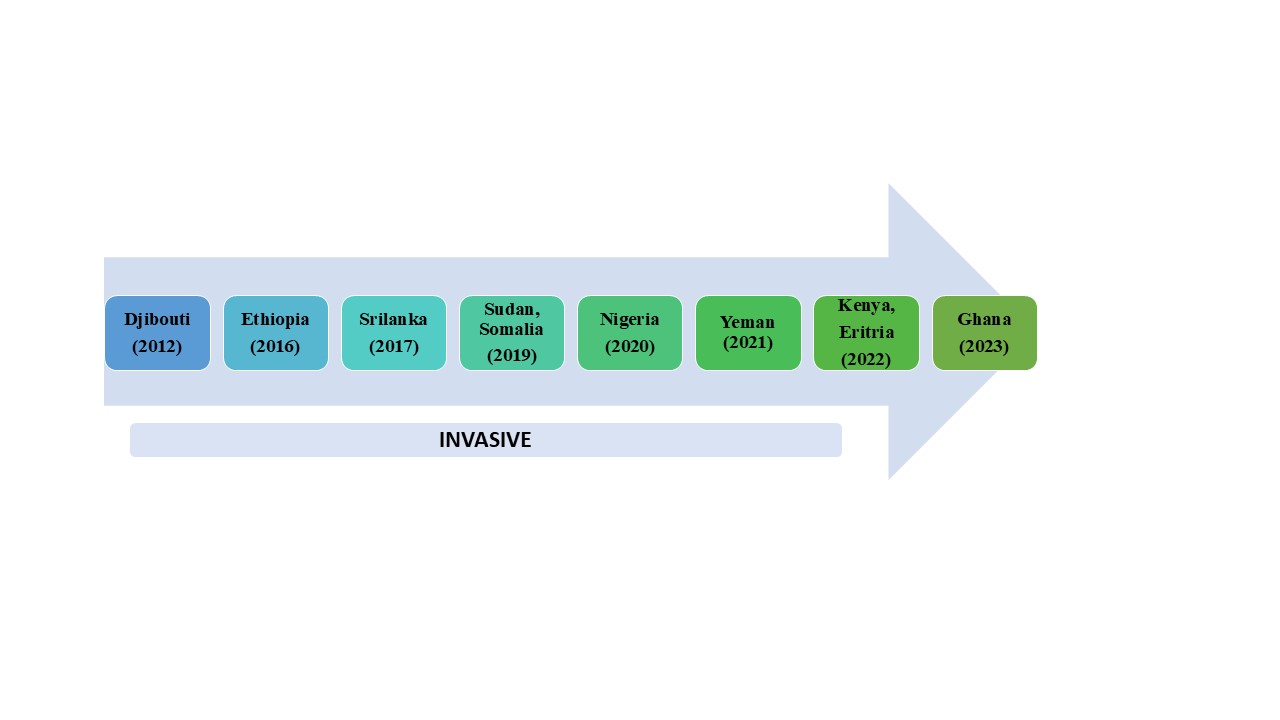
**
