# Supplementary material for: Topic: Distribution of Anopheles stephensi bioforms in selected districts of Rajasthan, India
Source: PLoS One. 2025 Feb 21;20(2):e0313227. doi: 10.1371/journal.pone.0313227 (PMC11844831; doi:10.1371/journal.pone.0313227)
Supplement: S1 Table — (DOCX) [file pone.0313227.s002.docx]

**S1 Table : Crossbreeding Experiment between *Anopheles stephensi* bioforms.**

| **Experiment** | **No of eggs laid** | **Ridge range** | **Emergence** | **Pupae** |
| --- | --- | --- | --- | --- |
| 4♂ Mysorensis X 2 ♀ Type | 151 | 14 to 18 | 137 | 125 |
| 4♂ Type X 2 ♀ Mysorensis | 140 | 13 to 18 | 124 | 112 |

**S2 Table : Morphometric analysis of *An. stephensi* bioform Egg ridges from different locations**

| **Districts** | **Locality type** | **Localities** | **Average RC** | **Mysorensis** | **Intermediate** | **Type** | **Total** |
| --- | --- | --- | --- | --- | --- | --- | --- |
| Barmer | Urban | Barmer | 16.41 | 20 | 78 | 151 | 249 |
|  | Rural | Baitu | 14.294 | 394 | 138 | 83 | 615 |
|  |  | Goliya | 15.87 | 5 | 17 | 10 | 32 |
|  |  | Gunga | 14.82 | 20 | 14 | 12 | 46 |
|  |  | Jasol | 15.18 | 34 | 44 | 16 | 94 |
|  |  | kalyanpura | 17.89 | 11 | 40 | 149 | 200 |
|  |  | Kanasar | 18.08 | 0 | 8 | 38 | 46 |
|  |  | Kawas | 14 | 72 | 26 | 5 | 103 |
|  |  | Nimbala | 13 | 10 | 0 | 0 | 10 |
|  |  | Rani | 15.4 | 2 | 6 | 2 | 10 |
| Bikaner | Urban | Biakner | 16.8 | 0 | 4 | 16 | 20 |
|  | Rural | Bhanipura | 14.88 | 11 | 10 | 4 | 25 |
|  |  | Diyatra | 14.8 | 6 | 8 | 1 | 15 |
|  |  | Kakku | 15.9 | 6 | 4 | 10 | 20 |
|  |  | kakra | 18.22 | 7 | 13 | 119 | 139 |
|  |  | Khara | 14.37 | 39 | 25 | 31 | 95 |
|  |  | Kodamdesar | 12.21 | 14 | 0 | 0 | 14 |
|  |  | Naal | 14.3 | 7 | 3 | 0 | 10 |
|  |  | Napasar | 15.5 | 1 | 7 | 2 | 10 |
| Jaipur | Urban | Jaipur | 14.86 | 34 | 24 | 16 | 74 |
| Jaisalmer | Urban | Jaisalmer | 13.85 | 7 | 0 | 0 | 7 |
|  | Rural | Away | 18.75 | 0 | 0 | 4 | 4 |
|  |  | Chinnu | 11.28 | 7 | 0 | 0 | 7 |
|  |  | Gomath | 15.58 | 8 | 18 | 10 | 36 |
|  |  | hamira | 13.52 | 19 | 2 | 0 | 21 |
|  |  | Indranagar | 16 | 0 | 9 | 1 | 10 |
|  |  | Raghwa | 14.43 | 32 | 21 | 0 | 53 |
|  |  | Raimala | 17 | 0 | 1 | 9 | 10 |
|  |  | Ramdevra | 16.58 | 12 | 21 | 34 | 67 |
|  |  | satyaya | 15.07 | 29 | 32 | 15 | 76 |
|  |  | That | 13.92 | 25 | 11 | 3 | 39 |
|  |  | Veeramdevra | 17.73 | 0 | 7 | 23 | 30 |
| Jodhpur | Rural | Jhalamand | 11.4 | 269 | 21 | 5 | 295 |
|  |  | Mahadev Nagar | 17.93 | 0 | 0 | 15 | 15 |
|  |  | Mandor | 13.83 | 10 | 2 | 0 | 12 |
|  | Urban | Bamba | 12.84 | 32 | 0 | 0 | 32 |
|  |  | kheme ka Kua | 14.42 | 67 | 33 | 13 | 113 |
|  |  | Public Park | 14.01 | 424 | 200 | 20 | 644 |
|  |  | Shastri Circle | 12.2 | 30 | 0 | 0 | 30 |
|  |  | University | 13.36 | 22 | 0 | 0 | 22 |
| Pali | Rural | Kharda | 12.83 | 12 | 0 | 0 | 12 |
|  | Urban | Pali | 14.71 | 6 | 8 | 0 | 14 |
| Sanchore | Rural | Jalore | 17.51 | 2 | 19 | 41 | 62 |
| Udaipur | Urban | Udaipur | 14.62 | 69 | 39 | 23 | 131 |
| Total Rural | | | 15.25 | 1072 | 527 | 642 | 2241 |
| Total Urban | | | 14.37 | 711 | 386 | 239 | 1336 |
| Total | | | 14.97 | 1064 | 943 | 843 | 3569 |
